# Effects of a Single Oral Dose of Imepitoin on Veterinary Visit-Related Stress in Cats: An Open-Label Pilot Study

**DOI:** 10.3390/vetsci13050431

**Published:** 2026-04-28

**Authors:** Umut Burak Agan, Estelle Descout, Yucel Meral

**Affiliations:** 1 Department of Internal Medicine, Faculty of Veterinary Medicine, Ondokuz Mayıs University, Samsun 55139, Türkiye; 2 Department of Ethology, Physiology and Chemical Communication, Institute of Research in Semiochemistry and Applied Ethology (IRSEA), 84400 Apt, France; 3 Statistics and Data Management Service, Institute of Research in Semiochemistry and Applied Ethology (IRSEA), 84400 Apt, France

**Keywords:** imepitoin, cat, veterinary visit, stress, behaviour

## Abstract

Stress associated with veterinary visits represents a significant concern for cats, as well as a primary challenge for both caregivers and veterinary practitioners. Based on previous in vitro and in vivo studies on imepitoin, this pilot study was conducted to explore its potential as an alternative treatment for stress associated with veterinary visits in cats. Cats were randomly allocated to receive imepitoin (*n* = 16) or placebo (*n* = 16) and were then evaluated using pre- and post-treatment physiological parameters, including heart rate (HR), respiratory rate (RR), rectal temperature (RT), salivary cortisol and behavioural scores derived from the cat stress score (CSS) and cat examination response scale (CERS). Despite higher baseline HR in the imepitoin group, a greater reduction in HR from pre- to post-treatment was observed compared to placebo. RR decreased, and RT slightly increased over time, regardless of treatment. A differential pre–post change in cortisol was observed between groups, with a significant decrease in the imepitoin group. Behavioural scores demonstrated improvement in the imepitoin-treated group; however, observer bias inherent in the open-label design, together with the use of a single investigator for both treatment administration and outcome assessment, limits the interpretability of the behavioural findings. These exploratory observations suggest a potential role for imepitoin in mitigating clinic-associated stress in cats; however, confirmation in larger, well-controlled, blinded, and methodologically robust studies is required before any clinical application can be considered.

## Simple Summary

Veterinary visits can be highly stressful for cats and may make examinations more challenging for both veterinarians and caregivers. In this context, the present pilot study explored whether imepitoin could help reduce stress in cats during veterinary visits. A total of 32 cats were randomly allocated to two groups: one group received imepitoin and the other received a placebo. Several indicators of stress were measured before and after treatment in a clinical setting, including heart rate, respiratory rate, rectal temperature, salivary cortisol levels, and behavioural scores during the veterinary examination. Although a baseline imbalance was observed for heart rate, cats that received imepitoin showed reductions in heart rate and salivary cortisol levels following treatment, which may suggest lower stress levels. Respiratory rate decreased, and rectal temperature increased slightly over time in both groups, regardless of treatment. Behavioural scores also improved in the imepitoin group, although observer bias cannot be ruled out due to the open-label design. Overall, the findings provide preliminary support for a potential role of imepitoin in the management of veterinary visit-related stress in cats. However, given the study’s methodological limitations, these results are insufficient to support direct clinical use and warrant further evaluation in well-designed, adequately powered controlled studies.

## 1. Introduction

Stress associated with veterinary visits represents a significant concern for feline patients, as well as a primary challenge for both caregivers and veterinary practitioners [1]. Feline stress directly affects caregivers’ attitudes and feline access to veterinary care, while simultaneously compromising staff safety and the quality of examinations [1,2]. Accordingly, effective management of feline stress is essential for maintaining high standards of clinical care.

Mitigating stress associated with veterinary visits in feline patients may require a multimodal approach that integrates environmental modifications, specialised personnel training, pheromones and pharmacological interventions [3,4,5]. Research supports the efficacy of pre-visit pharmacological agents such as gabapentin and trazodone in reducing stress and anxiety, although dose-dependent adverse effects are reported [6,7,8,9]. In addition, gabapentin may interfere with neurological assessment, thereby confounding clinical examination findings [10]. A comprehensive overview of pharmacological pre-visit anxiolytics is provided elsewhere [3].

Imepitoin, originally developed as a partial benzodiazepine-receptor agonist for the treatment of epilepsy in humans, was subsequently introduced into veterinary medicine owing to species-specific pharmacokinetic properties [11]. Imepitoin acts at the benzodiazepine binding site of the gamma-aminobutyric acid type A (GABA-A) receptor with low affinity and partial agonist activity, producing anxiolytic effects without significant sedation [12,13]. In vitro evidence demonstrates suppression of corticotropin-releasing factor-induced neuronal activity within the locus coeruleus without altering basal activity, suggesting stress attenuation independent of global neuronal depression [12]. In dogs, imepitoin has been shown to reduce noise-induced anxiety while maintaining a favourable safety profile [14,15]. Following oral administration, effective plasma concentrations are reached within 30 min in dogs, with peak levels occurring at 2–3 h [16]. A similar pharmacokinetic profile has been reported in cats, with peak plasma concentrations occurring within 1–3 h and good tolerability at doses up to 80 mg/kg, without major adverse effects or elevations in hepatic enzyme activity [17]. Although imepitoin demonstrates anxiolytic properties in dogs and has shown favourable pharmacokinetic and safety profiles in cats, its efficacy in mitigating acute situational stress in cats remains unexplored. Addressing this gap is essential to determine whether imepitoin has the potential to serve as an alternative or adjunct to existing pharmacological interventions. In light of this, this pilot study aimed to assess the potential of a single oral (PO) dose of imepitoin to reduce stress associated with veterinary visits in cats and to determine whether its use for this novel indication warrants further investigation.

## 2. Materials and Methods

### 2.1. Animals

The study subjects were client-owned healthy cats (*n* = 32) presented to the vaccination and antiparasitic treatment unit of the Ondokuz Mayis University Animal Hospital and enrolled voluntarily. Following pre-treatment assessment, cats were enrolled in the study if they met the following inclusion criteria: aged 1–7 years, confirmed to be healthy based on clinical examination and a comprehensive medical and behavioural history, and no administration of psychoactive substances within the preceding month. Additionally, only cats reported by caregivers to exhibit stress during previous veterinary visits, as manifested by behaviours such as hiding, fearful posture, vocalisation, tachypnoea, or elimination, and presenting a CSS ≥ 3 at pre-treatment, were included.

### 2.2. Experimental Design

**Figure 1:**
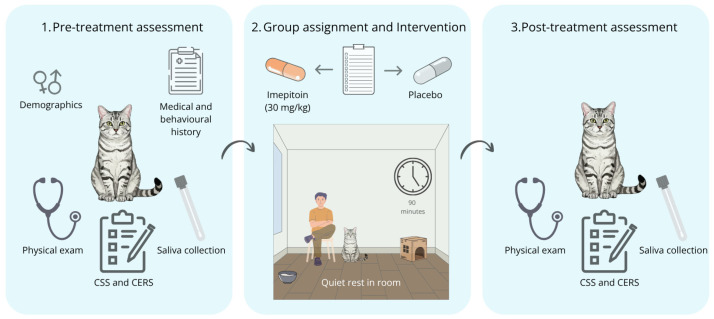
The schematic illustrates the three phases of the experimental protocol in cats. (1) Pre-treatment assessment: collection of demographic data, medical and behavioural history, physical examination, completion of behavioural scales (CSS and CERS) and saliva sampling, respectively. (2) Group assignment and intervention: cats were randomly assigned to receive imepitoin (30 mg/kg) or placebo, followed by a 90 min period of quiet rest in a room with their owner present. (3) Post-treatment assessment: repeat physical examination, CSS and CERS evaluations and saliva collection to assess treatment-related changes.

A power analysis was conducted prior to the study using G*Power 3.1 (version 3.1.9.4) to determine the required sample size. Based on Stevens et al. [8], a sample size of 32 subjects was estimated to provide 82% power to detect a significant difference between groups at an alpha level of 0.05. The study was designed as a randomised, placebo-controlled, open-label trial. An open-label design was adopted to enable close monitoring of potential adverse effects associated with imepitoin administration in this initial evaluation for this indication while also addressing practical constraints related to clinical workflow and personnel availability. Consequently, the same veterinarian administered treatment and performed all clinical assessments. The protocol consisted of three sequential phases, as illustrated in Figure 1. During the pre-treatment assessment, demographics and a detailed medical and behavioural history, including the method of transport to the clinic, were recorded. All cats enrolled in the study were examined by the attending veterinarian, who had no prior clinical interaction with them. Each cat underwent a standardised physical examination and behavioural assessment lasting approximately 15–20 min, followed immediately by saliva collection. A pre-generated simple randomisation list comprising 32 allocations (imepitoin, *n* = 16; placebo, *n* = 16) was created using an online list randomiser (random.org) prior to study initiation. Subjects meeting the inclusion criteria were subsequently allocated to treatment groups according to this randomisation list. The intervention consisted of a single dose of imepitoin (Pexion^®^, Boehringer Ingelheim, Ingelheim, Germany, 100 mg, 30 mg/kg, PO) or a placebo gelatin capsule. The selected dose was based on a previous study demonstrating its efficacy and safety in treating epilepsy in cats, suggesting sufficient GABAergic activity and a low incidence of adverse effects at this dose [17]. Treatments were administered manually at the end of saliva collection and 90 min before the post-treatment evaluation to approximate expected near-peak plasma concentrations of imepitoin during post-treatment clinical assessment. During this interval, cats remained with their caregivers in a quiet, standardised room, with free movement and access to a transport box, water, and litter. After the 90 min period, cats and caregivers returned to the examination room for a post-treatment assessment, during which the physical examination, behavioural assessment, and saliva sampling were repeated in full, as per the initial protocol.

### 2.3. Standardised Physical Examination

To ensure procedural consistency, all cats underwent a standardised physical examination conducted by the same veterinarian in a uniform environment using a nose-to-tail approach. The examination was performed in a consistent chronological sequence. Cats were first placed on the table in open carriers for three minutes to allow voluntary exit; if necessary, they were then gently removed by the veterinarian. Caregivers remained present throughout the assessment. Individual body weights were subsequently recorded using a calibrated electronic balance. Clinical parameters were then evaluated, including capillary refill time, hydration status, mucous membrane colour, and lymph node palpation. Finally, heart rate (HR) and respiratory rate (RR) were documented in beats per minute (bpm) and breaths per minute (brpm), respectively, via thoracic auscultation with a Littmann^®^ Classic stethoscope (Columbia, MO, USA) and rectal temperature (RT; °C) was recorded with a silicone-bodied electronic thermometer.

### 2.4. Behavioural Assessments

**Figure 2:**
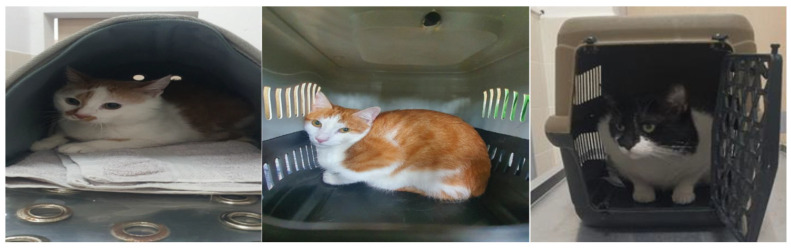
Representative images of cats included in the study, with Cat Stress Scores (CSS) of 5, 4, and 3 (from left to right).

Approximately 3 min after the physical examination, cats were assessed using two behavioural scales. Stress was quantified with the Cat Stress Score (CSS), an 11-variable behavioural scale ranging from 1 (relaxed) to 7 (terrified), with the final score defined as the modal value across variables [18]. Representative examples are presented in Figure 2. The scale is a well-established, validated instrument with nearly three decades of use and has shown good to excellent intra- and inter-rater reliability across multiple independent studies [19,20]. Handling tolerance was evaluated using the Cat Examination Response Scale (CERS), a 5-point scale adapted from a previous study [21], where 1 indicates proactive acceptance, and 5 indicates that examination is not possible due to resistance (Table 1). Behavioural scoring was performed by the first author, a doctoral candidate in veterinary medicine with a clinical focus on stress and behavioural disorders, who has prior experience in the routine application of the instruments and familiarity with their operational definitions and scoring criteria.

### 2.5. Saliva Collection and Cortisol Analysis

Saliva was collected using sterile swabs (Fıratmed^®^, Ankara, Türkiye) rubbed along the buccal mucosa and sublingual area for 30 s, with two samples obtained per cat. Samples were centrifuged at 100× *g* for 5 min. The recovered saliva was transferred into alphanumerically labelled Eppendorf tubes and stored at −20 °C following Yozova et al. [22]. Specimens were transported on dry ice to an independent laboratory, where cortisol (COR) concentrations were quantified using the Cat Cortisol COR ELISA Kit (BT-Lab^®^, Shanghai, China) and measured with a microplate reader in accordance with the manufacturer’s instructions. All samples were analysed in a single run to minimise inter-assay variation. The assay sensitivity was 0.28 ng/mL, with intra- and inter-assay coefficients of variation of <8% and <10%, respectively. Two discoloured placebo samples with insufficient volume were excluded from analysis.

### 2.6. Statistical Analysis

Statistical analyses were conducted using the R Statistical language software (version 4.4.3) on RStudio 2024.12.1 Build 563 © 2009–2025 Posit Software, PBC, Boston, MA, USA. The significance threshold was fixed at the classical value of 5%. Missing data (saliva samples unavailable for two individuals in the placebo group) were coded as NA and excluded from the analyses. Demographic characteristics were compared using the Mann–Whitney U test or Fisher’s exact test, as appropriate. Continuous variables were analysed using General Linear Mixed Models (GLMMs). Conditions of residue normality (graphically and using normality tests) and homoscedasticity (“Residuals versus fits” graphic and dispersion test) were verified and validated. Discrete variables related to scores of cats were analysed thanks to Mixed Ordinal Logistic Regression models (MOLR). The proportional odds assumption was assessed using the nominal test. For all models, fixed effects included treatment group (Placebo vs. Imepitoin), time (Pre vs. Post) and their interaction (treatment group x time). Subject (cat) was included as a random effect to account for within-subject repeated measures. Gender and neutered status were added to models as covariates. Multiple comparisons were performed using the Tukey test to identify significant effects with more than two modalities. Complete models were simplified if the AIC and BIC criteria decreased when a factor was removed from models (and if this one was not significant). Effect sizes for statistically significant continuous outcomes were reported as Cohen’s *d* together with their corresponding 95% confidence intervals.

## 3. Results

### 3.1. Study Population and Demographics

The study population comprised 32 subjects, with a mean age of 2.58 ± 1.59 years. The median age was 2.25 years, and ages ranged from 1 to 7 years. The study included 21 males (65.6%) and 11 females (34.4%). Most cats were neutered (*n* = 25, 78.1%) and were Domestic Shorthairs (*n* = 24, 75.0%). Demographic characteristics, including age, sex, breed, neuter status and mode of transportation, were comparable between the study groups (*p* > 0.05 for all comparisons) and are presented in Table 2.

### 3.2. Descriptive Statistics

Continuous variables (HR, RR, RT, and COR) were summarised as mean ± standard deviation for each treatment group at pre- and post-treatment (Table 3). Ordinal behavioural scores (CSS and CERS) were summarised as counts and percentages for each category by treatment group and time point (Table 4).

### 3.3. Physical Examination and Physiological Variables (HR, RR and RT)

In all cats included in the study, inspection and palpation findings were normal. Capillary refill time was ≤2 s, and palpable lymph nodes were normal. Furthermore, no abnormal findings were detected on thoracic auscultation.

A significant group-by-time interaction was observed for HR (*p* = 0.0022), indicating differential pre–post change between treatment groups. Although pre-treatment HR values were higher in the imepitoin group compared with placebo (*p* = 0.0076), HR significantly decreased in the imepitoin group between pre- and post-treatment (Δ = −29.62, *p* < 0.0001). In contrast, no significant change was observed in the placebo group (Δ = −4.31, *p* = 0.4667). The difference in pre–post change between groups was −25.3, corresponding to a large treatment effect (Cohen’s *d* = −1.08, 95% CI −1.85 to −0.31; Table 5).

A significant main effect of time was observed for RR (*p* = 0.0098), indicating that RR decreased from pre- to post-measurements (Pre: 65.66 ± 26.13; Post: 58.88 ± 21.10) regardless of group. RR decrease (Δ = −6.78) represented a small effect size (Cohen’s *d* = −0.27, 95% CI −0.49 to −0.06; Table 5). Similarly, a significant main effect of time was also observed for RT (*p* = 0.0127), indicating that RT increased from pre- to post-measurements (Pre: 38.58 ± 0.34; Post: 38.71 ± 0.36). RT increase (Δ = +0.13) corresponded to a small effect size (Cohen’s *d* = 0.36, 95% CI 0.06 to 0.66; Table 5).

### 3.4. Saliva Cortisol (COR)

A significant group-by-time interaction was observed for COR (*p* = 0.0254), indicating differential pre–post change between treatment groups. COR significantly decreased in the imepitoin group between pre- and post-treatment (Δ= −0.66, *p* = 0.0324), whereas no significant change was observed in the placebo group (Δ = 0.30, *p* = 0.3477). The difference in pre–post change between groups was −0.96, corresponding to a large treatment effect (Cohen’s *d* = −0.82, 95% CI −1.60 to −0.04; Table 5).

### 3.5. Behavioural Assessments (CSS and CERS)

Given the open-label design and the use of a single observer for outcome assessment, the following behavioural findings should be interpreted with appropriate caution. A significant group-by-time interaction was observed for CSS (*p* = 0.0004), indicating differing trajectories between groups. CSS decreased markedly from pre- to post-treatment in the imepitoin group, with minimal change in the placebo group. Post-treatment scores differed significantly between groups (*p* = 0.0044), and a significant pre–post reduction occurred within the imepitoin group (*p* < 0.0001), with a visible shift to lower scores (Table 4 and Table 5). Similarly, CERS showed a significant group-by-time interaction (*p* < 0.0001), reflecting differential change over time. The imepitoin group demonstrated marked pre–post improvements, whereas placebo changes were smaller. Between-group differences were significant at both pre-treatment (*p* = 0.0394) and post-treatment (*p* = 0.0011), with a significant within-group improvement for imepitoin (*p* < 0.0001).

### 3.6. Adverse Effects

No adverse effects related to imepitoin or placebo administration were observed during in-clinic monitoring. According to post-visit owner reports in the imepitoin group, mild adverse effects were noted in six cats (37.5%), including increased sleepiness with reduced appetite in three cats (18.8%) and reduced appetite alone in three cats (18.8%). Six cats (37.5%) did not exhibit any adverse effects. Follow-up information could not be obtained for the remaining four cats (25.0%) in the imepitoin group. No post-visit owner-reported adverse event data were collected for the placebo group.

## 4. Discussion

This pilot study aimed to evaluate the potential role of imepitoin in mitigating veterinary-visit-related stress in cats and presents preliminary evidence that a single oral dose (30 mg/kg) of imepitoin may warrant further evaluation, given the observed treatment-related effects in cats under the conditions of this study. To the best of our knowledge, this study is one of the first evaluations of imepitoin for the mitigation of situation-induced stress in cats, addressing a clinically important yet under-researched area. Demographic and transport-related variables were largely comparable between groups, reducing the likelihood that these factors substantially influenced the observed outcomes. However, male cats were overrepresented in both groups. Previous evidence suggesting that male cats may vocalise more during veterinary visits raises the possibility that behavioural responses to clinic-associated stress could vary by sex [23]. Furthermore, because only adult cats were included, the extent to which these findings apply to younger or geriatric populations, where stress responses may differ, remains uncertain. Several other potential sources of confounding should be considered. In particular, prior veterinary experiences may represent an important confounding factor. None of the enrolled cats had previously visited the attending veterinarian; all had experienced at least one prior veterinary visit unrelated to this study, which was subjectively described as stressful by their owners. Although this inclusion criterion may have reduced heterogeneity among the study subjects to some extent, no detailed data were collected on the frequency, nature, or valence of these prior experiences. Consequently, variability in learned associations with veterinary environments may have influenced both behavioural and physiological responses observed in this study [23,24]. In addition, individual temperament and environmental factors during transport are other possible confounders. Together, these variables highlight the multifactorial nature of feline stress responses and warrant cautious interpretation of the treatment effects.

Certain physiological and behavioural parameters in cats, such as HR, RR, cortisol responses, and stress scores, are known to be influenced by veterinary visits [24]. In this study, the most notable physiological change associated with imepitoin was observed in HR. Although baseline HR was higher in the imepitoin group, differences in temporal patterns between groups may suggest an effect on the cardiovascular response to examination. HR reflects sympathetic activation and is a recognised physiological stress biomarker in felines [24,25,26]. The magnitude of HR reduction in this study parallels the “white coat effect” described by Quimby et al. [27], who reported a mean stress-related increase of 33 bpm, supporting the interpretation of reduced examination-related stress in imepitoin-treated cats. Physiologically, the inhibitory effects of GABA-A receptor activation on cardiovascular responses, particularly via sympathetic modulation in the dorsomedial periaqueductal grey, may underlie imepitoin’s ability to suppress stress-induced HR elevations, consistent with its partial agonist activity at GABA-A receptors [13,28]. However, given the higher baseline HR values in the treatment group, the observed reduction should be interpreted with caution, as it may be partly attributable to baseline differences or regression to the mean. Thus, while the direction of change is suggestive, the clinical relevance of the observed HR reduction remains uncertain and requires confirmation in studies with balanced baseline values and additional cardiovascular measures. RR and RT exhibited only modest temporal variation and showed no treatment-specific modulation. RR is recognised as a parameter positively associated with acute stress in feline patients, particularly in clinical settings [29,30]. Despite the small effect size, the significant decrease in RR observed in both groups suggests a treatment-independent reduction in physiological stress, most likely attributable to habituation and repeated exposure to the experimental procedure and environment. Furthermore, mean RR values in the present study (Pre: 65.66 ± 26.13; Post: 58.88 ± 21.10) were consistent with previous reports from clinical settings, including median values of 70–73 breaths/min (range 46–105) and 58 breaths/min (range 18–192), indicating comparable stress conditions and supporting marked inter-individual variability in RR among cats [27,31]. Unexpectedly, RT increased significantly over time irrespective of treatment, although the change was clinically negligible (Δ = 0.13 °C). This minor increase may be attributable to the study methodology, particularly increased physical activity during the resting period, as the cats were allowed to move freely. It may additionally reflect the effects of prolonged time spent in the clinical environment. In a previous study comparing rectal temperature between home and hospital environments in cats, the observed increase in RT was considered negligible and was not statistically or clinically significant [27]. Together, these findings suggest that multiple variables influence RT and should be interpreted cautiously in the context of clinic-related stress in cats. The absence of treatment-specific effects may further indicate that RR and RT might be less responsive to pharmacological anxiolysis under these conditions. Importantly, the order of clinical measurements may have affected the observed physiological responses. As HR was assessed before RR and RT, initial handling may have altered the cats’ physiological state, potentially affecting subsequent measurements. This potential order effect should be considered when interpreting these findings and when designing future studies.

Cortisol is widely used to assess physiological responses mediated by the hypothalamic–pituitary–adrenal (HPA) axis [32] and has been measured across many contexts and biological matrices in cats [33,34,35]. Although salivary cortisol is preferred for its non-invasive collection and practicality, research in cats remains limited [22,36,37,38]. Our results indicate that temporal changes differed between groups, with a significant reduction in salivary cortisol observed only in imepitoin-treated cats. The significant pre–post decrease and large between-group effect size suggest a clinically relevant treatment effect. At the same time, the absence of change in the placebo group supports a treatment-specific rather than nonspecific, temporal, or habituation-related explanation. These findings suggest that imepitoin may modulate the HPA axis sufficiently to reduce salivary cortisol levels, consistent with its known GABAergic mechanism of action and reported behavioural effects in other companion animals [12,13]. Furthermore, the cortisol response observed in the present study following imepitoin administration revealed changes in a similar direction to those reported in dogs. Imepitoin has been shown to markedly suppress the cortisol response in 81% of dogs exposed to thunderstorms in an open field [15]. Although decreased salivary cortisol may indicate a reduced stress response, it is generally recommended to corroborate this finding with behavioural observations and other parameters [39]. In the present study, improvements were observed in both stress scores (CSS) and examination response (CERS); however, given the unblinded, single-investigator design, the behavioural outcomes are particularly susceptible to expectation and detection bias. As both scales rely on subjective interpretation, there is a substantial risk that treatment effects are overestimated. In one blinded study, pre-visit administration of gabapentin was associated with a significantly lower CSS (mean difference of −1.65) as assessed by owners compared with placebo [9]. In this context, the directional consistency of our behavioural findings with those reported for gabapentin may lend support to the clinical plausibility of imepitoin. However, given the methodological limitations of our study, no strong conclusions can be drawn regarding the magnitude of the effect or causality.

In the present study, imepitoin appeared generally well-tolerated, with no serious adverse effects observed under the study conditions. The absence of serious adverse effects in dogs also indirectly supports the safety profile of imepitoin [15]. Although other anxiolytic agents, including gabapentin, trazodone, and benzodiazepines, have been associated with a range of adverse effects in previous reports [3], direct comparisons should be made with caution because of differences in study design, populations, and outcome measures. Furthermore, because follow-up data were unavailable for a subset of cats, the frequency and nature of adverse effects in this study should be interpreted with caution. Previous work has shown that following oral administration of imepitoin at 40 and 80 mg/kg in cats, the most frequently reported adverse effect was increased salivation, followed by a mild reduction in food intake [17]. In the present study, the absence of serious adverse effects is consistent with these findings; however, the lack of observed salivation is noteworthy and may be related to the tablet administration method or the dose used. Nevertheless, longer-term follow-up in larger patient populations, including monitoring of haemato-biochemical parameters, is warranted to characterise the safety profile of imepitoin more comprehensively and to determine the clinical relevance and potential cumulative nature of any adverse effects over time.

Current scientific data on the pharmacological management of veterinary visit-related stress in cats remains limited, with gabapentin and trazodone the principal options [3,7,8,9,10]. Gabapentin appears clinically effective and has an acceptable safety profile; however, most studies rely on behavioural outcomes without supporting physiological or endocrinological markers. In addition, its sedative and analgesic effects may mask pain-related signs [9] and influence clinical assessments, particularly neurological examinations [10], highlighting the need to evaluate alternatives such as imepitoin in specific examination contexts. Trazodone has similarly demonstrated efficacy in reducing transport- and clinic-related stress, although the available evidence is constrained by small sample sizes [8]. A similar study evaluating transdermal trazodone reported promising reductions in anxiety and stress; however, it was also limited by a small sample size (*n* = 13) and a lack of veterinarian blinding [7]. In this study, imepitoin was administered in a clinical setting, providing an initial reference for its use in stressed cats. Future studies should evaluate home administration before clinic visits, as earlier dosing before transport and related stressors may enhance efficacy. Overall, differences in study design, including drug administration timing, assessment approaches, and blinding, limit direct comparisons and support the consideration of clinical trials comparing gabapentin and/or trazodone with imepitoin to establish comparative efficacy.

This pilot study has several limitations that warrant careful consideration when interpreting the findings. First and foremost, the absence of blinding due to open-label design may have introduced observer bias, as outcome assessments were conducted with knowledge of the study conditions. This lack of masking increases the risk that subjective assessment methods, such as CSS and CERS, were influenced by prior expectations. Nevertheless, behavioural outcomes were assessed using predefined, standardised scales designed to maximise objectivity and consistency across observations. Objective physiological parameters were also included to provide convergent evidence and strengthen overall reliability. Second, the study was conducted by a single investigator, which may have introduced multiple sources of bias, including observer, measurement, and detection bias. Additionally, certain potential confounding factors were not adequately controlled. In particular, the cats’ prior clinical experiences may have influenced their responses during the study, thereby affecting the observed outcomes. Beyond assessment-related considerations, the relatively small sample size may have reduced statistical power for outcomes characterised by high biological variability. Accordingly, while the findings offer valuable preliminary insights, they should be considered exploratory rather than confirmatory and should not be used to inform clinical decision-making. Taken together, these limitations highlight the need for future studies incorporating larger sample sizes, appropriate blinding procedures, multiple investigators, and more rigorous control of confounding variables to strengthen the validity and reliability of the conclusions.

## 5. Conclusions

This pilot study presents preliminary findings supporting the potential role of a single oral dose of imepitoin (30 mg/kg), administered 90 min before a veterinary examination, in mitigating stress in cats. Although methodological limitations may have affected the reliability of behavioural outcomes, the direction of the behavioural changes, together with changes in objective parameters such as heart rate and cortisol levels, suggests a potential treatment-related effect. These findings do not permit definitive conclusions; however, they support further investigation in larger, controlled, and methodologically robust studies and should not be used for clinical decision-making at this stage.

## Figures and Tables

**Table 1:** Cat Examination Response Scale (CERS), adapted from Pereira et al. [21].

Score		Description
1	Accepts physical examination	The cat approaches the veterinarian, seeks contact, and displays friendly behaviour (tail held upright, ears semi-back, slow blinking, whiskers relaxed to the sides). The physical examination is performed effortlessly.
2	Physical examination easily performed	The cat does not approach the veterinarian but shows no resistance during handling and allows the examination to be performed without difficulty.
3	Reluctant to undergo physical examination	The cat is restless, may attempt to escape or hiss when approached; however, the examination can be completed with mild restraint.
4	Physical examination difficult	The cat tries to escape and may growl, hiss, strike, or bite when the veterinarian approaches. The examination requires firm restraint and the use of protective equipment.
5	Physical examination impossible	The cat becomes extremely aggressive, cannot be controlled, and physical examination is not possible.

**Table 2:** Comparison of sociodemographic characteristics of the imepitoin and placebo groups.

Variable		Imepitoin (*n* = 16)	Placebo (*n* = 16)	Statistical Test	*p* Value
Age	Mean ± SD	2.22 ± 1.32	2.94 ± 1.80	Mann–Whitney U Test (Wilcoxon)	0.2413
	Median (min-max)	2.00 (1–5)	2.75 (1–7)		
Sex	Female, *n* (%)	4 (25)	7 (44)	Fisher’s Exact Test	0.4578
	Male, *n* (%)	12 (75)	9 (56)		
Breed	Domestic shorthair, *n* (%)	12 (75)	12 (75)	Fisher’s Exact Test	1.0000
	British shorthair, *n* (%)	1 (6)	2 (13)		
	Scottish fold, *n* (%)	1 (6)	2 (13)		
	Norwegian forest cat, *n* (%)	1 (6)	0 (0)		
	Maine coon, *n* (%)	1 (6)	0 (0)		
Neuter status	Neutered, *n* (%)	13 (81)	12 (75)	Fisher’s Exact Test	1.0000
	Intact, *n* (%)	3 (19)	4 (25)		
Transport	Private vehicles, *n* (%)	9 (56)	7 (44)	Fisher’s Exact Test	0.7244
	Public transportation, *n* (%)	7 (44)	9 (56)		

SD: Standard Deviation, min: minimum, max: maximum.

**Table 3:** Descriptive statistics of continuous variables in placebo and imepitoin groups pre and post-treatment.

Variable	Time	Placebo (*n* = 16)	Imepitoin (*n* = 16)
HR (bpm)	Pre	140.31 ± 24.17	165.50 ± 26.72
	Post	136.00 ± 18.88	135.88 ± 30.73
RR (brpm)	Pre	62.56 ± 33.01	68.75 ± 17.34
	Post	56.88 ± 22.13	60.88 ± 20.54
RT (°C)	Pre	38.56 ± 0.30	38.60 ± 0.39
	Post	38.61 ± 0.34	38.81 ± 0.36
COR (ng/mL)	Pre	2.31 ± 1.60	2.77 ± 1.59
	Post	2.61 ± 1.32	2.11 ± 1.36

Sample size was *n* = 16 per treatment group, except for cortisol measurements in the placebo group (*n* = 14) due to excluded samples.

**Table 4:** Distribution of CSS and CERS scores pre- and post-treatment.

Variable	Score	Pre *n* (%)		Post *n* (%)	
		Placebo	Imepitoin	Placebo	Imepitoin
CSS	1	0 (0.00)	0 (0.00)	0 (0.00)	1 (6.25)
	2	0 (0.00)	0 (0.00)	1 (6.25)	8 (50.00)
	3	6 (37.50)	6 (37.50)	7 (43.75)	5 (31.25)
	4	8 (50.00)	4 (25.00)	6 (37.50)	1 (6.25)
	5	2 (12.50)	5 (31.25)	2 (12.50)	0 (0.00)
	6	0 (0.00)	1 (6.25)	0 (0.00)	1 (6.25)
CERS	1	1 (6.25)	0 (0.00)	0 (0.00)	7 (43.75)
	2	9 (56.25)	8 (50.00)	9 (56.25)	6 (37.50)
	3	6 (37.50)	6 (37.50)	6 (37.50)	2 (12.50)
	4	0 (0.00)	2 (12.50)	1 (6.25)	1 (6.25)

Values are presented as *n*, number (%).

**Table 5:** Results of GLMMs and MOLR evaluating the effects of treatment, time and their interaction, including multiple comparisons.

Variable	Effect	Comparison	χ^2^ (df)	*p*-Value	Cohen’s *d* [95% CI]
HR	Treatment × Time		9.36 (1)	0.0022	−1.08 ^†^ [−1.85, −0.31]
		Imepitoin vs. Placebo (pre)	-	0.0076	-
		Imepitoin (post vs. pre)	-	<0.0001	-
RR	Time		6.67 (1)	0.0098	−0.27 [−0.49, −0.06]
RT	Time		6.20 (1)	0.0127	0.36 [0.06, 0.66]
COR	Treatment × Time		5.00 (1)	0.0254	−0.82 ^†^ [−1.60, −0.04]
		Imepitoin (post vs. pre)	-	0.0324	-
CSS	Treatment × Time		12.78 (1)	0.0004	-
		Imepitoin vs. Placebo (post)	-	0.0044	-
		Imepitoin (post vs. pre)	-	<0.0001	-
CERS	Treatment × Time		44.00 (1)	<0.0001	-
		Imepitoin vs. Placebo (pre)	-	0.0394	-
		Imepitoin vs. Placebo (post)	-	0.0011	-
		Imepitoin (post vs. pre)	-	< 0.0001	-

All reported *p*-values are statistically significant. ^†^ Large effect size. CI, Confidence Interval. vs., Versus.

## Data Availability

The data collected during this study are available from the corresponding author upon request but are not publicly available as they form part of a PhD thesis.
